# Intramedullary Spinal Cord Metastasis as Initial Presentation of Malignant Melanoma: A Unique Case Report and Role of Contrast vs Non-contrast MRI in Its Diagnosis

**DOI:** 10.7759/cureus.19731

**Published:** 2021-11-18

**Authors:** Abdul Manan, Syed Rizvi, Jyothi Kondlapudi

**Affiliations:** 1 Nephrology and General Internal Medicine, The Royal Wolverhampton NHS Trust, Wolverhampton, GBR; 2 Acute and General Internal Medicine, University Hospitals Birmingham, Birmingham, GBR

**Keywords:** sox 10, human melanoma black 45, rim and flame sign, malignant melanoma initial presentation, spinal cord compression, contrast enhanced mri, conus medullaris syndrome, iscm, intramedullary spinal cord metastasis, malignant melanoma metastasis

## Abstract

Intramedullary spinal cord metastasis (ISCM) is a diagnostically challenging and dreadful complication of cancer. Twenty-seven cases of ISCM exclusively related to malignant melanoma have been reported so far in a recent study.On review of literature, we could not find any reported case with ISCM secondary to malignant melanoma as initial presentation. To the best of our knowledge, we are reporting the first such case. We report a case of a 71-year-old lady presenting with gradual onset of bilateral leg weakness “off legs” and lower limb paresthesias. On examination she had an upper motor neuron pattern lower limb weakness with reduced sensations to all modalities and brisk reflexes with extensor plantar responses. She was evaluated with non-contrast MRI (magnetic resonance imaging) spine which showed focal myelopathic cord signal at the conus and at the level of T10 and T11 vertebrae (radiological differential diagnosis given on MRI were B12 deficiency/inflammatory/infection). Thorough radiological scans were ordered which revealed a disseminated malignancy. A biopsy sample from gastric lesion revealed diagnosis of malignant melanoma. A repeat MRI whole spine with gadolinium contrast was done later with suspicion of spinal metastasis which has led to lower limb weakness. MRI with contrast showed an enhancing soft tissue metastatic mass lesion within conus in comparison with plain MRI done one week earlier. At present, diagnostic modalities available for diagnosing ISCM particularly secondary to melanoma do not have high specificity. Contrast MRI is the diagnostic modality of choice at present. Non-contrast MRI has low sensitivity in diagnosis of ISCM compared to contrast MRI and could potentially delay the management, especially in highly aggressive malignancies like malignant melanoma where an early diagnosis and treatment is critical for better outcome.

## Introduction

Intramedullary spinal cord metastasis (ISCM) is a diagnostically challenging and disastrous complication of cancer. ISCM secondary to metastatic melanoma is rare; however, the number of such cases is showing an increasing trend [1,2] probably due to newer treatment modalities of malignant melanoma leading to prolonged survival rates in this malignancy [3,4]. ISCM occurring as the initial presentation of malignant melanoma is an extremely rare presentation and till now no such case has been reported in literature. The extradural lesions account for up to 95% of spinal metastasis of all malignancies. Intradural extramedullary and intramedullary metastasis of systemic cancer account for 5-6% and 0.5-1% of spinal metastases, respectively [5]. In terms of diagnosis of ISCM, neuroimaging does not consistently show a standardized pattern, however MRI is the imaging tool of choice. Usually, the confirmatory diagnosis of spinal melanoma is only made based on post-surgical pathological studies or autopsies [6]. We are reporting a case of malignant melanoma with lower limb motor and sensory symptoms as initial presentation secondary to ISCM. The unique occurrence of intramedullary spinal cord metastasis as the initial presentation and the absence of pathognomic symptoms in ISCM combined with low diagnostic sensitivity on non-contrast MRI led to diagnostic delay in our patient.

## Case presentation

**Figure 1:**
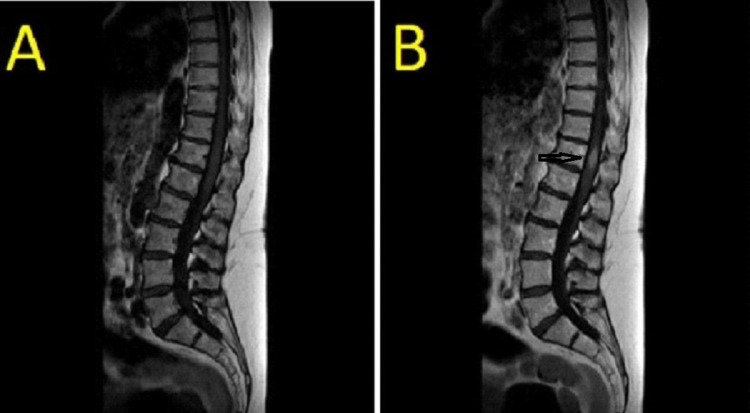
MRI non-contrast (A), MRI with gadolinium contrast showing “flame sign” (B)

A 71-year-old lady initially presented with gradual onset of painless lower limb weakness for one week “off legs”, lower limb paresthesias and increased urinary frequency. Patient denied any recent symptoms of infective etiology. There was no recent or past history of trauma or accidents. Patient’s past medical history was unremarkable and also had no family history of significant concern. Patient was a non-smoker and teetotaler and there was no exposure to environmental factors such as industrial chemicals, radiation, heavy metals or any toxin exposures reported. Patient led an active life as a housewife (Eastern Cooperative Oncology Group [ECOG] performance status 1 before admission) and used to live with her partner. On examination she had symmetrical lower limb motor weakness in an upper motor neuron pattern with Medical Research Council (MRC) muscle power 4/5 both proximally and distally on admission. There were reduced sensations in lower limbs symmetrically to fine touch, pinprick, temperature and proprioception, initially mild but progressed to significant sensory loss over next couple of weeks with sensory level slightly below umbilicus at T11. There was relative sparing of saddle area and anal tone was patulous. There was no spinal tenderness. Her deep tendon reflexes in lower limbs were exaggerated with extensor plantar responses. Rest of the general and systemic examination was unremarkable. Her blood tests including full blood count, inflammatory markers, B12, folate, thyroid functions, calcium, liver function test and myeloma screen came back normal. Cerebrospinal fluid (CSF) analysis done on second day after admission was unyielding and non-specific with normal protein, glucose and cytology etc. She was evaluated with non-contrast MRI spine which showed focal myelopathic cord signal at the conus and at the level of T10 and T11 vertebrae (radiological differential diagnosis on MRI were B12 deficiency/inflammatory/infection) (Figure 1A).

While being evaluated for same, she underwent a chest x-ray which was suggestive of suspicious nodules in the right lower and mid zone. This triggered further computed tomography (CT) scans with contrast which showed disseminated malignancy with metastatic lesions to anterior chest wall, liver, lungs, and a necrotic 3.5 cm nodal mass in the left gastric region. There was no obvious primary identified on the CT scan. Tumor markers cancer antigen (CA)-125, CA 19-9, carcinoembryonic antigen (CEA) and CA 15-3 were all negative. A repeat MRI spine with contrast was done afterward with suspicion of spinal metastasis which has led to lower limb weakness. MRI contrast showed a 20*10*19 mm enhancing soft tissue metastatic mass lesion seen within conus in comparison with plain MRI done one week earlier (Figure 1B).

**Figure 2:**
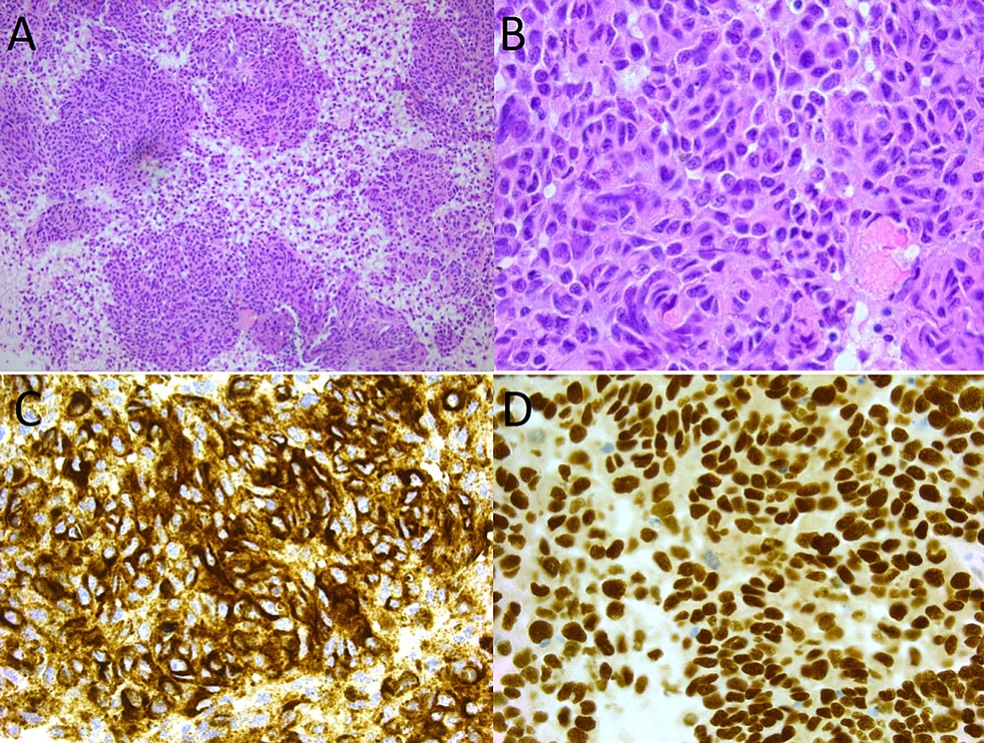
EUS FNA sample of perigastric mass: H&E X10 (A), H&E X40 (B), HMB45 X40 (C), SOX10 X40 (D) EUS: Endoscopic ultrasound, FNA: Fine needle aspiration, H&EX10: Haematoxylin and eosin stain with 10 times magnification, H&E X40: Haematoxylin and eosin stain with 40 times magnification, HMB45 X40: Human melanoma black 45 stain with 40 times magnification, SOX10 X40: (Sry-related HMg-Box gene 10) stain with 40 times magnification.

There was no obvious primary identified on the CT. This was discussed in upper GI MDT (Gastrointestinal multidisciplinary team) and was thought to be a disseminated malignancy arising from a gastric primary. An endoscopic ultrasound (EUS) showed a deep, malignant-looking gastric ulcer on the high anterior greater curve of stomach. This was seen with EUS as a hypoechoic mass extending into the serosal margin. Biopsy from the stomach lesion surprisingly showed a metastatic malignant melanoma. Histopathology showed scanty strips of benign columnar epithelium with most of the tissue representing tumour, focally necrotic (Figure 2). Immunohistochemical assays for focal Melan-A, human melanoma black-45 and SOX10 confirmed the diagnosis of malignant melanoma. Real-time polymerase chain reaction (PCR) analysis of the BRAF gene was done which revealed the presence of a mutation within codon 600.

This case was discussed again in dermatology-oncology MDT after biopsy results and was decided that the best course of action would be palliative treatment, considering the advanced stage of the disease and poor performance status. Management options and prognosis were discussed with patient who agreed to palliative management. After discharge patient contracted coronavirus disease 2019 (COVID-19) pneumonia for which she was again hospitalized and treated with oral dexamethasone 6mg (milligrams) once a day and oxygen inhalation via nasal cannula between 2-4lit/min for 10 days. She further had developed bilateral segmental pulmonary emboli and was started on anticoagulation with apixaban. Her neurological symptoms worsened and progressed to paraplegia, urinary and fecal incontinence over a period of three to four weeks since initial presentation. Patient passed away three months after diagnosis of metastatic melanoma under community palliative care.

## Discussion

Intramedullary spinal cord metastasis is an uncommon presentation of malignancies. Autopsy series have reported that 0.9 to 2.1% of all patients with cancer exhibit intramedullary spinal cord metastases [7]. ISCM secondary to malignant melanoma account for 2% of all cases of ISCM. In the literature, ISCM of malignant melanoma is described as a small percentage of the ISCMs of all cancer types; thus, it is sometimes not reported in detail. Twenty-seven cases of ISCM of malignant melanoma have been reported so far in a recent study [8]. On review of literature, we could not find any reported case with ISCM secondary to malignant melanoma as initial presentation. To the best of our knowledge, we are reporting the first such case.

Diagnosing ISCM of malignant melanoma has been challenging. It has been suggested that MRI is the most effective imaging technique for revealing spinal cord melanoma. According to most authors, the MRI pattern of spinal cord melanoma includes signal hyperintensity on T1-weighted images and signal iso- or hypo-intensity on T2-weighted images [8]. With injection of an appropriate contrast medium, there should be a moderate enhancement of the lesion [9]. On contrast-enhanced MRI, two unique highly specific characteristics of ISCMs, rim and flame signs, have been reported. Rim sign indicate a more intense thin rim with peripheral enhancement than other tumor areas and flame signs indicate flame-shaped enhancements at the edge of the lesion. Depending upon the variable degree of tumour melanin content, the signal characteristics on MRI are biased [8,10-12]. Flame and rim sign, two unique characteristics of ISCM on contrast, are present in a much lower percentage in ISCM of other cancers [8]. Considering the above facts, it is diagnostically challenging to detect ISCM of melanoma. While evaluating our patient we did a non-contrast MRI initially which was not able to detect malignant intramedullary soft tissue mass within the conus medullaris and misinterpreted it as focal myelopathic cord signal. Repeat MRI with contrast was needed to reveal enhancing soft tissue mass measuring 22*10*19 mm. Definitive diagnosis of ISCM of melanoma is only made by histopathological studies on biopsy samples or autopsies [6,13-15]. Immunochemical studies also aid in the diagnosis [16]. S-100 protein, focal Melan-A, SOX10 and human melanoma black-45 are used in diagnosis. In our patient, biopsy sample from gastric metastasis was taken which supported malignant melanoma as histopathology and immunochemical staining for focal Melan-A, SOX10 and human melanoma black-45 were positive.

Surgery remains the treatment of choice for localized melanoma. For metastatic melanoma besides chemotherapy, newer treatment modalities like oncogene and immune-targeted strategies have been developed over last decade. These patient-centered treatment options, based on specific markers and mutations, are a swiftly growing field and the increased knowledge of molecular targets and drugs tailored accordingly [17]. Although they have demonstrated remarkable efficacy in some patients, their effect on overall survival is still variable [18]. After having multidisciplinary team discussions we believed our patient was ineligible for checkpoint inhibitor immunotherapy or targeted therapy with combination of BRAF and MEK (Mitogen-activated protein kinase) inhibitors and the best course of action available was palliative management.

## Conclusions

Neurological symptoms secondary to ISCM could be the initial presentation of metastatic malignancy. This is an extremely rare case report of a patient with malignant melanoma with symptoms secondary to ISCM as initial presentation. At present, diagnostic modalities available for diagnosing ISCM particularly secondary to melanoma does not have high specificity. Contrast MRI is the diagnostic modality of choice at present. Non-contrast MRI has low sensitivity in diagnosis of ISCM compared to contrast MRI and could delay the management especially in highly aggressive malignancies like malignant melanoma where early diagnosis and treatment is critical for better outcome. Furthermore, malignant melanoma with ISCM is an advanced disease state and eventually causes paraparesis/quadriparesis, urinary and/or faecal incontinence, which can lead to poor functional status. At this advanced stage of the disease, palliative treatment involving multidisciplinary team can help to keep the patient comfortable and improve the quality of life, while offering best supportive care to balance symptom control versus life sustaining measures based on patient’s priorities.
